# Phytosterol Contents of Edible Oils and Their Contributions to Estimated Phytosterol Intake in the Chinese Diet

**DOI:** 10.3390/foods8080334

**Published:** 2019-08-09

**Authors:** Ruinan Yang, Li Xue, Liangxiao Zhang, Xuefang Wang, Xin Qi, Jun Jiang, Li Yu, Xiupin Wang, Wen Zhang, Qi Zhang, Peiwu Li

**Affiliations:** 1 Oil Crops Research Institute, Chinese Academy of Agricultural Sciences, Wuhan 430062, China; 2 Key Laboratory of Biology and Genetic Improvement of Oil Crops, Ministry of Agriculture and Rural Affairs, Wuhan 430062, China; 3 Laboratory of Quality and Safety Risk Assessment for Oilseed Products (Wuhan), Ministry of Agriculture and Rural Affairs, Wuhan 430062, China; 4 Quality Inspection and Test Center for Oilseed Products, Ministry of Agriculture and Rural Affairs, Wuhan 430062, China; 5 Key Laboratory of Detection for Mycotoxins, Ministry of Agriculture and Rural Affairs, Wuhan 430062, China

**Keywords:** phytosterols, vegetable oils, foods, China, dietary intake

## Abstract

Phytosterols are important micronutrients in human diets. Evidence has shown that phytosterols play an essential role in the reduction of cholesterol in blood and therefore decrease cardiovascular morbidity. In this study, the content and composition of phytosterols in different kinds of vegetable oils were analyzed, and the total phytosterol intake and contribution of foods to intake were estimated based on consumption data. The results showed that the phytosterol contents of rice bran oil, corn oil, and rapeseed oil were higher than those of other vegetable oils and the intake of phytosterol in the Chinese diet was about 392.3 mg/day. The main sources of phytosterols were edible vegetable oils (46.3%), followed by cereals (38.9%), vegetables (9.2%), nuts (2.0%), fruits (1.5%), beans and bean products (1.4%), and tubers (0.8%). Among all vegetable oils, rapeseed oil was the main individual contributor to phytosterol intake (22.9%), especially for the southern residents of China.

## 1. Introduction

Phytosterols are plant-derived sterols that have similar physiological functions with cholesterol in vertebrate animals [1,2]. More than 100 types of phytosterols and 4000 other types of triterpenes have been found according to the literature [3]. β-Sitosterol, campesterol, stigmasterol, brassicasterol, and ∆5-avenasterol are the main phytosterols in plants [4]. Phytosterols exist in different forms in plants, including free or esterified with fatty acids, steryl glycosides, and acylated glycosides [5]. In recent years, phytosterols have attracted increasing interest due to their hypocholesterolemic capacity and potential contribution to a decreased risk of cardiovascular diseases [6,7]. Previous studies have indicated that phytosterols could decrease cardiovascular morbidity by reducing cholesterol absorption through different mechanisms, which play a crucial role in the pathogenesis of dementia via the pathway [8,9]. Katan et al. reported that the intake of 2 to 3 g of phytosterols per day was essential to significantly reduce both the total cholesterol and low-density lipoprotein (LDL)-cholesterol levels in the blood by 10% [10]. Furthermore, phytosterols also have some other health-promoting effects, such as anti-inflammatory [11], immunomodulatory [12], and anticancer effects [13]. Food and Agriculture Organization of the United Nations (FAO), National Institutes of Health (NIH), and other authorities have advised normal or hypercholesterolemic persons to uptake an adequate amount of phytosterols to lower cholesterol levels [14]. FAO has suggested that the acceptable daily intake (ADI) is 0–40 mg/kg of body weight (bw) for phytosterols, phytostanols, and their esters. Commission Regulation (European Union (EU)) No. 686/2014 states that a beneficial effect can be obtained with a daily intake of 1.5–3 g of plant sterols/stanols. For the Chinese population, the recommended amounts for phytosterols and phytosterol esters put forward in Chinese Dietary Reference Intakes (2013 Edition) are 0.9 g/day and 1.5 g/day, respectively [15].

In the past decades, the Chinese diet has changed from being rich in plant-derived foods to being rich in animal source foods [16]. This dietary change led to a decrease in phytosterol intake and an increase in cholesterol intake. A higher intake of cholesterol is correlated with an increased cardiovascular disease risk and the diet poses a threat to human health [17]. It was found that a higher intake of phytosterol from the diet was significantly associated with lower concentrations of the total and serum LDL-cholesterol [18]. Therefore, it is necessary to investigate and review the dietary sources of phytosterols for Chinese and to find potential foods to help improve their phytosterol intake.

The intakes of phytosterol from dietary sources in Western countries and Mediterranean countries have been reviewed previously [19,20,21,22]. It was found that the range of phytosterol intake was 150–450 mg/day and vegetable oils such as corn oil, rapeseed oil, and sunflower oil were regarded as the richest dietary sources of phytosterols [23]. Cereals, vegetables, fruits, and nuts also made important contributions to phytosterol intake [24,25].

At present, there are various types of vegetable oils in the Chinese market, including soybean oil, rapeseed oil, peanut oil, and sunflower oil, which occupy a major portion. Meanwhile, there are some minor vegetable oils emerging in the Chinese market, such as corn oil, camellia oil, and peony oil, to meet the various consumer demands. In the import market, olive oil and rice bran oil are mainly imported from Spain and Italy. These vegetable oils all have many well-known brands and are obtained by different oil processing technologies. It is well known that the oil extraction method, refining degree, and material source affect the content and composition of phytosterols [26,27,28,29]. The purpose of this study was to investigate the phytosterol contents of different kinds of vegetable oils. Then, the phytosterol intake of the Chinese diet was estimated based on the consumption data of plant-derived foods obtained from the report of the Nutrition and Health Status of Chinese Residents [30]. In addition, the contributions of plant-derived foods and vegetable oils were also investigated. The study of the main contributors to phytosterol intake in the Chinese diet may be very helpful in dietary intake guidance.

## 2. Materials and Methods

### 2.1. Data Source

The data on the percentage of every oil intake to total vegetable oil intake and the consumption of plant-derived foods were obtained from the United States Department of Agriculture (USDA) [31] and the report of the Nutrition and Health Status of Chinese Residents [30]. This study used the database containing the contents of phytosterols (β-sitosterol, campesterol, stigmasterol, β-sitostanol, and campestanol) in the plant source foods commonly consumed in China. The database included more than 160 food items, and the data on the contents of phytosterols in vegetables and fruits had previously been published [32].

### 2.2. Oil Samples

Vegetable oils (10 peanut oils, 14 soybean oils, 14 rapeseed oils, 15 sesame oils, 14 olive oils, 10 camellia oils, 15 sunflower oils, 15 corn oils, 7 rice bran oils, 10 flaxseed oils, 3 walnut oils, 3 grapeseed oils, and 3 peony seed oils) were all commercial oils collected from the markets of different provinces. The oil samples were of different brand and grade. For example, sunflower oils were of different types, such as first grade pressed oil, refined oil, and fragrant oil. Detailed information for the oil samples is listed in Table S1, Supplementary Materials. The oil samples were stored in darkness at 4 °C for further analysis.

### 2.3. Phytosterol Analysis

The standard samples of brassicasterol, ergosterol, campesterol, campestanol, stigmasterol, β-sitosterol, ∆5-avenasterol, cycloartanol, cycloartenol, 24-methylene-cycloartanol, and cholestanol were purchased from Sigma-Aldrich Chemical Co. (St Louis, MO, USA). The standard solutions were prepared by dissolving a known amount of each standard in acetone to reach the concentration of 1 mg/mL. The cholestanol standard solution was used as an internal standard solution in this study.

A 50 mg of oil was placed in a sealable tube with 200 μL internal standard solution. Alkaline hydrolysis was performed by adding 5 mL of 2 M KOH in ethanol, and the mixture was shaken and heated at 75 °C for 30 min. After saponification, the tube was cooled to room temperature. Then, 2 mL of distilled water and 5 mL of hexane were added and the mixture was shaken thoroughly to extract unsaponifiable matter. The unsaponifiable matter was extracted three times with hexane. Then, the hexane fractions were combined and evaporated under a stream of nitrogen. 100 μL N-methyl-N-trimethylsilylheptafluorobutyramide-1-methylimidazole (95:5, *v/v*) mixture was added to the residual sterol. Afterward, the vial was sealed and heated at 75 °C for 20 min, then cooled to room temperature. Hexane was added to complete the volume to 1 mL before GC-MS analysis.

Sterols were analyzed by a Shimadzu GC-MS TQ-8040 (Shimadzu Corp., Kyoto, Japan) equipped with a DB-5 MS capillary column (30 m × 0.25 mm × 0.25 μm, Agilent Technologies, Palo Alto, CA, USA). The injection volume was 1 μL in spilt mode at a ratio of 20:1. The carrier gas was helium with a flow rate of 1.2 mL/min. Analyses were performed under the following temperature program: oven temperature increased from 100 to 290 °C at a rate of 40 °C/min and held for 15 min at 290 °C. The temperatures of ion source and transfer line were 250 °C and 290 °C, respectively. Scan was performed by selected ion monitoring mode (SIM), and the quantitative and qualitative ions for each compound were selected based on previous literature [33]. Sterols were identified by comparing their mass spectra and retention times with the corresponding standards. The phytosterol contents of oil samples were calculated based on the standard curves obtained by internal standard method with cholestanol as the internal standard.

### 2.4. Statistical Analysis

Every oil sample was tested three times and all results were recorded as mean value ± standard deviation. The comparison of the values was evaluated by one-way analysis of variance and Duncan’s test using IBM SPSS Statistics version 20.0 (SPSS Inc., Chicago, IL, USA), and all results were considered significant at the level of *p* < 0.05.

## 3. Results

### 3.1. Phytosterol Compositions of Different Vegetable Oils

**Figure 1:**
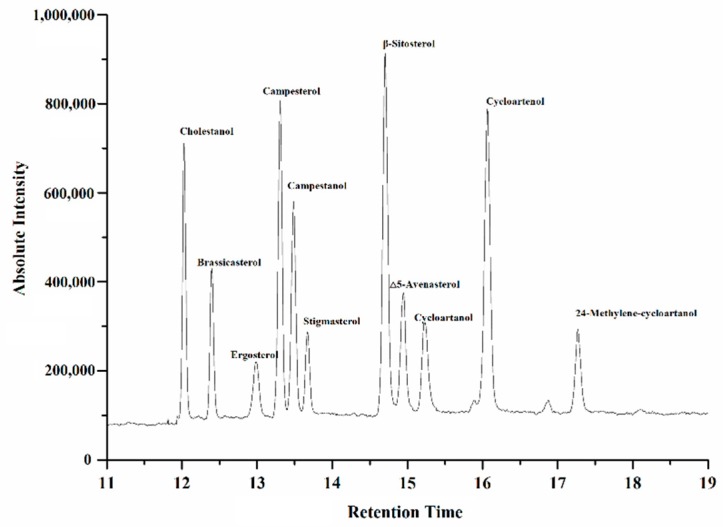
Total ion chromatogram of silylated sterol standards by GC-MS analysis.

The phytosterol compositions of the vegetable oils were analyzed by GC-MS. Satisfactory separation for cholestanol, brassicasterol, ergosterol, campesterol, stigmasterol, β-sitosterol, ∆5-avenasterol, campestanol, cycloartanol, cycloartenol, and 24-methylene-cycloartanol were obtained by GC-MS, as shown in Figure 1.

Phytosterol compositions of different vegetable oils are shown in Table 1 and phytosterol contents of vegetable oils are expressed as mg/100 g. It can be seen that β-sitosterol, campesterol, stigmasterol, and ∆5-avenasterol were the main phytosterols for all kinds of vegetable oils. As the predominant phytosterols, β-sitosterol and campesterol contents accounted for more than 50% of total phytosterol contents except for camellia oil, in which they accounted for 46.69%. Cycloartanol took up a larger proportion of total phytosterols in camellia oil than other oils. Compared with other vegetable oils, rice bran oil had higher contents of every kind of phytosterol expect brassicasterol, which was found predominantly in the Brassicaceae family. Rapeseed oil, as the production of rape, contained a certain amount of brassicasterol with a percentage of 15.29%. A small amount of brassicasterol was also found in soybean oil with a percentage of 3.60%. Soybean oil also had a higher content of stigmasterol, which could reduce the risk of ovarian, esophageal, and ovarian cancers [34]. Compared with other vegetable oils, rice bran oil and corn oil had higher contents of stanols (campestanol, cycloartanol, and 24-methylene-cycloartanol). Stanols, the saturated forms of phytosterols, are considered a subgroup of phytosterols. It has been reported that phytostanols occur in trace levels in many plant species but in high levels in only a few cereal species [35]. This was consistent with the result of this study. As shown in Table 1, the data for phytosterol had a higher standard deviation, which may be due to the differences of material, extraction method, and refining degree. It has been reported that vegetable oils extracted using different methods showed different composition and content of phytosterols [26,27]. During oil refining steps, such as degumming, neutralization, bleaching, and deodorization, the oil showed a loss of phytosterols, especially for the bleaching and deodorization processes [28,29]. In order to obtain an oil with a higher content of phytosterols, appropriate measures have been taken to retain these compounds, and sunflower and rice bran oils with higher contents of phytosterols are available on the market.

### 3.2. Total Phytosterol Contents of Different Vegetable Oils

The total phytosterol contents, the sum of the contents of the above phytosterols, of different vegetable oils are shown in Table 1. The total phytosterol contents of the vegetable oils ranged between 142.64 and 1891.82 mg/100 g. Higher phytosterol contents were detected in rice bran oils (1891.82 mg/100 g), corn oil (990.94 mg/100 g), and rapeseed oil (893.84 mg/100 g). The results were in line with Verleyen’s report [4]. The phytosterol contents of sesame oil and flaxseed oil were 637.60 mg/100 g and 466.73 mg/100 g, respectively. Soybean oil, peanut oil, and olive oil were similar in phytosterol content (approximately 300 mg/100 g). The phytosterol content of sunflower oil was 253.25 mg/100 g, which was higher than the limit (100 mg/100 g) suggested by the European Union (EU). In comparison with these vegetable oils, the content of phytosterols in camellia oil (142.64 mg/100 g) and palm oil (150.00 mg/100 g) [36] was relatively low. It might suggest that phytosterol contents of herbal oils were higher compared with the phytosterol contents of wood oils. It can be seen that the phytosterol contents of different vegetable oils varied greatly. Furthermore, the phytosterol contents varied obviously even for the same kind of oil. The phytosterol contents of rice bran oils varied from 1351.43 mg/100 g to 2842.48 mg/100 g. The phytosterol contents of corn oils were in the range of 510.17 mg/100 g to 1433.65 mg/100 g. The phytosterol contents of rapeseed oils were in the range of 558.34 mg/100 g to 1406.87 mg/100 g. For other oils, the maximum phytosterol contents were also more than twice the minimum. This may be a result of variations in genetic species, growing and storage conditions, refining processes, and analytical methods [26,27,28,29].

The mean concentration of phytosterols in vegetable oils can be calculated based on the percentage of edible oils consumed in China. Phytosterol contents of these oils are listed in Table 2, and the mean concentration is 486.66 mg/100 g.

## 4. Discussion

### 4.1. Intake of Plant Source Foods and Phytosterols in the Chinese Diet

Phytosterols existed in all plant source food items and the contents of phytosterols varied with the plant food group. From Table 3, it can be seen that vegetable oils contained higher phytosterols than cereals, beans and bean products, vegetables, and other plant-derived foods, as reported in Ostlund’s paper [37]. The overall intake of phytosterol from the Chinese diet was about 392.3 mg/day, which fell into the range mentioned by Ostlund (167–437 mg/day) [37]. The intake of phytosterol in the Spanish and Dutch diets were, respectively, 375 mg/day and 285 ± 97 mg/day [19,20]. In Finland, the intake of phytosterols was 305 mg/day for men and 237 mg/day for women [21]. Compared with these countries, China has a higher intake of phytosterol. Because phytosterols exist in many foods and the contents of phytosterols vary with food groups, the variations in food patterns may influence the total intake of phytosterol [20]. However, it was still inadequate and less than the recommended amounts (0.9 g/day for phytosterols and 1.5 g/day for phytosterol esters) put forward in Chinese Dietary Reference Intakes (2013 Edition) published by the Chinese Nutrition Society [15].

According to Table 3, it can be seen that the main contributors to the total phytosterol intake among all plant food groups were vegetable oils (46.3%), cereals (38.9%), vegetables (9.2%), followed by nuts (2.0%), fruits (1.5%), beans and bean products (1.4%), and tubers (0.8%). Vegetable oil contribution was significant because of its higher phytosterol concentration and larger amount of consumption. A higher intake of phytosterol may be associated with a higher consumption of vegetable oils. Cereals and vegetables were not rich in phytosterols, but were also important dietary sources of phytosterols due to the higher amounts consumed. Nuts, rich in phytosterols (202.35 mg/100 g), were often eaten in trace amounts, but could still contribute to the total phytosterol intake [38].

**Figure 2:**
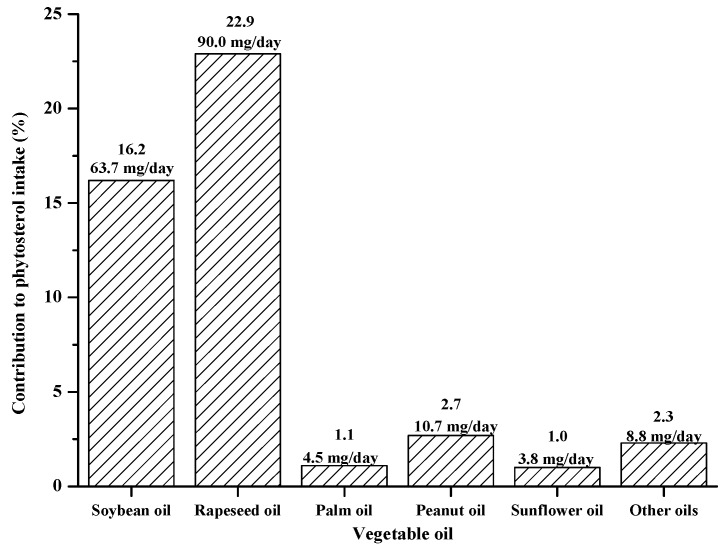
**Figure2.** Contributions of different vegetable oils to total intake of phytosterol in the Chinese diet.

In the Chinese diet, vegetable oils made the greatest contribution to the intake of phytosterol with a contribution percentage of 46.3%. Due to the dietary habits and cooking methods of Chinese people, this result may be slightly different from the Sweden and the Netherlands, which pointed out that cereals contributed more to the intake of phytosterol than vegetable oils as the consumption of the former was higher [39,40]. Based on the phytosterol concentrations of all kinds of vegetable oils and total oil intake listed in Table 2, the contribution of every kind of oil to the total phytosterol intake was calculated and is shown in Figure 2. For all kinds of vegetable oils, soybean oil and rapeseed oil were the two major ones, accounting for more than 75% of the total consumption. Rapeseed oil was the first domestic vegetable oil of China with the consumption ratio of 27%. It was also the main individual contributor to phytosterol intake and contributed 22.9% (90.0 mg/day) of the intake of phytosterol. The contributions of soybean oil, palm oil, peanut oil, and sunflower oil were 16.2% (63.7 mg/day), 1.1% (4.5 mg/day), 2.7% (10.7 mg/day), and 1.0% (3.8 mg/day), respectively. The total contribution of other oils was 2.3% (8.8 mg/day). It is worth mentioning that the intake of phytosterol was low for rice bran oil and corn oil in spite of their higher phytosterol concentrations. Compared with the Spanish diet [19], in which the main contributors were sunflower oil (52.7 mg/day) and refined olive oil (35.2 mg/day), rapeseed oil in the Chinese diet supplied more phytosterols. This may be due to several reasons. First, in the case of rapeseed oil, as the main edible vegetable oil in China, its consumption is higher. Second, the concentration of phytosterols in the same kind of oil may be different because of the varied breeds in different countries. The cultivar may have an effect on the contents of phytosterols in oils [41]. Third, the processing methods are not the same, as we know that extraction methods and refining processes influence the contents and compositions of phytosterols [42,43]. During the cooking process, phytosterols can be act as an antioxidant to prevent triacylglycerol polymer formation at frying temperature at the risk of degradation [44]. The degradation rate for phytosterols depends on the phytosterol structure, heat temperature, and time. The amount of phytosterol decreases as temperature and time increase, and the thermal stability is better for the phytosterol when there are fewer double bonds and a longer length of branch chain in the structure [33,45,46]. High temperature and short time are the main characteristics of typical Chinese cooking. The degradation rates for β-sitosterol, stigmasterol, campesterol, and brassicasterolat 180 °C for 15 min were 6%, 7%, 6%, and 8%, respectively [33]. It can be seen that cooking at a higher temperature and shorter time may have a slight effect on the phytosterol contents of oil. Given the maximum retention of phytosterols, a lower temperature and a shorter time should be adopted during cooking.

### 4.2. Intake of Plant Source Foods and Phytosterols for the Southern and Northern Residents of China

China is a multi-ethnic country, and different ethnic groups exhibit disparate lifestyles and eating habits. The predominant consumption of rice and rice products in South China versus flour and flour products in North China leads to a different intake of phytosterol. Due to higher concentrations of phytosterols in flour and flour products, the phytosterol intake for the northern residents (518.9 mg/day) was higher than that for the southern residents (345.9 mg/day), as listed in Table 4. In addition, the contribution of each plant food group was also different. For the southern residents, the main contributor group to total phytosterol intake was vegetable oils (51.2%). For the northern residents, the main contributor group to the total phytosterol intake was flour (40.2%), followed by vegetable oils (35.9%). Vegetable oils and cereals were the best natural sources of dietary phytosterols for both the southern and northern residents just as in other countries [47]. On the other hand, the major cultivars of rapeseed in China are mainly located in the Yangtze River Valley, such as Hubei, Sichuan, and Jiangsu Provinces. Therefore, the main consumption of vegetable oils in the south was rapeseed oil. Because of the higher phytosterol content of rapeseed oil (893.84 mg/100 g), as shown in Table 1, it may be inferred that the intake of phytosterol for the southern residents would be 494.2 mg/day if the concentration of rapeseed oil was substituted for the mean concentration of vegetable oils.

## 5. Conclusions

Phytosterols exist in vegetable oils with different concentrations and compositions, and rice bran oil, corn oil, and rapeseed oil have higher contents of phytosterols than other vegetable oils. Based on the data on food consumption and phytosterol contents in China, phytosterol intake was calculated as being 392.3 mg/day. Among the food groups, vegetable oils, especially rapeseed oil, were by far the greatest contributor to total phytosterol intake. However, Chinese Dietary Guidelines (2016) recommend that individuals should control the intake of oils to as low as 25–30 g/day for a balanced diet. On the basis of those guidelines, the intake of phytosterol may have declined. There is a need for Chinese people, especially the southern residents, to increase the consumption of oils with higher phytosterol concentrations to maintain and improve the intake of phytosterol. It is also necessary to increase the phytosterol contents in vegetable oils. To achieve this, research studies on vegetable oils should focus on processing methods aimed at reserving phytosterols in edible oils and the production of vegetable oils with high content of phytosterols.

## Figures and Tables

**Table 1:** Phytosterol Compositions of Different Vegetable Oils.

Vegetable Oil	Phytosterol Content (mg/100 g)										
	Brassicasterol	Egrosterol	Campesterol	Campestanol	Stigmasterol	β-Sitosterol	∆5-Avenasterol	Cycloartanol	Cycloartenol	24-Methylene-Cycloartanol	Total
Peanut oil	N.D.^1^	1.56 ± 1.28 ^a,b,2^	41.19 ± 9.82 ^d^	4.95 ± 2.15 ^c^	48.16 ± 13.33 ^c^	189.12 ± 42.40 ^d^	19.70 ± 9.74 ^d^	0.73 ± 0.82 ^b^	9.42 ± 4.35 ^c^	4.92 ± 1.32 ^c^	319.75 ± 76.15 ^d^
Soybean oil	12.80 ± 2.94 ^b^	1.98 ± 1.74 ^a,b^	62.68 ± 23.97 ^d^	5.91 ± 0.60 ^c^	87.28 ± 24.42 ^b^	166.03 ± 43.62 ^d,e^	7.21 ± 2.84 ^d^	2.72 ± 2.31 ^b^	4.71 ± 1.52 ^c^	4.34 ± 0.99 ^c^	355.67 ± 91.85 ^d^
Rapeseed oil	136.64 ± 66.34 ^a^	2.54 ± 1.92 ^a^	267.50 ± 61.24 ^a^	2.83 ± 1.71 ^c^	25.67 ± 8.99 ^c,d^	394.11 ± 146.74 ^c^	40.92 ± 29.58 ^c,d^	1.10 ± 0.96 ^b^	17.26 ± 14.43 ^c^	5.28 ± 1.60 ^c^	893.84 ± 237.77 ^b^
Sesame oil	N.D.	2.05 ± 2.49 ^a,b^	90.30 ± 32.78 ^c,d^	7.48 ± 8.94 ^c^	86.89 ± 32.77 ^b^	322.73 ± 85.81 ^c,d^	98.79 ± 42.35 ^b^	0.83 ± 1.12 ^b^	23.79 ± 19.14 ^c^	4.75 ± 1.21 ^c^	637.60 ± 180.59 ^c^
Olive oil	N.D.	0.83 ± 1.14 ^a,b^	25.85 ± 14.20 ^d^	1.61 ± 0.84 ^c^	21.13 ± 9.03 ^d^	152.05 ± 58.58 ^d,e^	29.73 ± 14.55 ^c,d^	2.79 ± 2.02 ^b^	19.44 ± 19.33 ^c^	34.58 ± 22.00 ^b,c^	288.02 ± 92.60 ^d^
Camellia oil	N.D.	2.62 ± 2.77 ^a^	16.52 ± 4.62 ^d^	0.22 ± 0.30 ^c^	22.11 ± 7.78 ^c,d^	50.09 ± 13.71 ^e^	1.81 ± 1.52 ^d^	29.11 ± 24.93 ^a^	17.84 ± 15.40 ^c^	2.33 ± 2.06 ^c^	142.64 ± 50.86 ^d^
Corn oil	4.13 ± 7.49 ^b^	0.87 ± 0.99 ^a,b^	197.32 ± 49.83 ^b^	74.53 ± 27.55 ^b^	45.53 ± 18.70 ^c,d^	539.93 ± 160.08 ^b^	97.92 ± 34.89 ^b^	1.89 ± 1.81 ^b^	15.85 ± 8.75 ^c^	12.97 ± 4.84 ^c^	990.94 ± 240.76 ^b^
Sunflower oil	0.58 ± 1.25 ^b^	0.25 ± 0.41 ^b^	28.36 ± 11.43 ^d^	1.71 ± 1.39 ^c^	18.69 ± 7.79 ^d^	170.91 ± 26.18 ^d^	12.45 ± 5.34 ^d^	0.18 ± 0.47 ^b^	8.85 ± 4.18 ^c^	11.27 ± 3.66 ^c^	253.25 ± 46.60 ^d^
Flaxseed oil	1.66 ± 2.91 ^b^	N.D.	115.52 ± 27.20 ^c^	4.17 ± 3.07 ^c^	12.62 ± 7.59 ^d^	157.79 ± 24.37 ^d,e^	56.01 ± 34.56 ^c^	0.99 ± 1.23 ^b^	78.67 ± 36.48 ^b^	39.29 ± 14.51 ^b,c^	466.73 ± 60.65 ^c,d^
Rice Bran oil	6.33 ± 3.04 ^b^	2.17 ± 1.01 ^a,b^	226.43 ± 85.91 ^a,b^	221.20 ± 79.47 ^a^	132.90 ± 55.47 ^a^	735.17 ± 185.99 ^a^	157.41 ± 72.12 ^a^	31.08 ± 16.86 ^a^	156.25 ± 93.73 ^a^	222.88 ± 115.87 ^a^	1891.82 ± 500.76 ^a^
Walnut oil	2.14 ± 0.54 ^b^	0.67 ± 0.19 ^a,b^	31.53 ± 18.66 ^d^	6.94 ± 5.26 ^c^	32.80 ± 19.08 ^c,d^	165.23 ± 69.07 ^d,e^	5.99 ± 7.40 ^d^	0.44 ± 0.62 ^b^	15.33 ± 7.38 ^c^	10.97 ± 4.55 ^c^	272.04 ± 107.41 ^d^
Peony oil	1.77 ± 0.30 ^b^	2.65 ± 1.47 ^a^	21.32 ± 14.30 ^d^	4.25 ± 6.01 ^c^	2.57 ± 2.23 ^d^	258.71 ± 18.45 ^d^	3.64 ± 4.16 ^d^	N.D.	6.37 ± 1.32 ^c^	65.90 ± 6.04 ^b^	367.19 ± 42.13 ^d^
Grapeseed oil	2.15 ± 0.93 ^b^	1.09 ± 0.26 ^a,b^	29.25 ± 5.21 ^d^	4.15 ± 2.31 ^c^	35.77 ± 3.97 ^c,d^	146.63 ± 14.67 ^d,e^	16.18 ± 7.89 ^d^	2.71 ± 2.52 ^b^	12.51 ± 6.46 ^c^	23.36 ± 11.18 ^c^	273.80 ± 38.85 ^d^

Data were recorded as mean values ± standard deviations. ^1^ N.D. represents not detected. ^2^ Different superscript letters within each column indicate a significant difference between each oil at the *p* < 0.05 level.

**Table 2:** Mean Concentration of Phytosterols of the Vegetable Oils.

Vegetable Oil	Percent of Total Vegetable Oil Intake (%)	Phytosterol Contents (mg/100 g)	Mean Concentration (mg/100 g)
Soybean oil	48 ^1^	355.67	486.66 ^2^
Rapeseed oil	27	893.84	
Palm oil	8	150.00	
Peanut oil	9	319.75	
Sunflower oil	4	253.25	
Rice bran oil	4	1891.82	
Corn oil		990.94	
Sesame oil		637.60	
Camellia oil		142.64	
Olive oil		288.02	
Flaxseed oil		466.73	
Walnut oil		272.04	
Grapeseed oil		273.80	
Peony seed oil		367.19	

^1^ The data on the percent of total intake of vegetable oils were obtained from the United States Department of Agriculture (USDA) [31]. ^2^ The mean concentration of phytosterols of the vegetable oil was the sum of the product of percent and phytosterol content for each vegetable oil. The data show that 4% was the total percent of nine kinds of vegetable oils.

**Table 3:** Intake of Plant Food and Phytosterol in the Chinese Diet.

Plant Food Group	Consumption (g/day)	Content of Total Phytosterols (mg/100 g)	Phytosterol Intake (mg/day)	Percent of Total Intake of Phytosterol (%)
Cereals	337.3 ^1^	45.23 ^2^	152.6	38.9
Tubers	35.8	8.43	3.0	0.8
Bean and bean products	10.9	50.67	5.5	1.4
Vegetables	269.4	13.42	36.2	9.2
Fruits	40.7	14.34	5.8	1.5
Nuts	3.8	202.35	7.7	2.0
Vegetable oils	37.3	486.66	181.5	46.3
Total			392.3	100.0

^1^ The consumption data of each plant food group were taken from the report of the Nutrition and Health Status of Chinese Residents [30]. ^2^ The total phytosterol contents for each group were obtained from the database containing the contents of phytosterols (β-sitosterol, campesterol, stigmasterol, β-sitostanol, and campestanol) in the plant source foods commonly consumed in China [32].

**Table 4:** Intakes of Plant Foods and Phytosterol between the Southern and Northern Parts of China.

Plant Food Group	Consumption (g/day)		Content of Total Phytosterols (mg/100 g)	Phytosterol Intake (mg/day)		Percent of Total Intake of Phytosterol (%)	
	North	South		North	South	North	South
Rice and rice products	72.60 ^1^	324.01	13.62 ^2^	9.9	44.1	1.9	12.7
Flour and flour products	349.96	44.64	59.60	208.6	26.6	40.2	7.7
coarse cereals	49.14	6.96	62.46	30.7	4.4	5.9	1.3
Tubers	14.61	13.7	8.43	1.2	1.2	0.2	0.3
Bean and bean products	17.38	46.12	50.67	8.8	23.4	1.7	6.8
Vegetables	282.48	339.43	13.42	37.9	45.6	7.3	13.2
Fruits	145.49	84.515	14.34	20.9	12.3	4.0	3.6
Nuts	7.24	5.52	202.35	14.7	11.2	2.8	3.2
Vegetable oils	38.27	36.4	486.66	186.2	177.1	35.9	51.2
Total				518.9	345.9	100	100

^1^ The consumption data of each plant food group was from the study undertaken by He [48]. ^2^ The total phytosterol contents for each group were obtained from the database containing the contents of phytosterols (β-sitosterol, campesterol, stigmasterol, β-sitostanol, and campestanol) in the plant source foods commonly consumed in China [32].

## Supplementary Materials

The following are available online at https://www.mdpi.com/2304-8158/8/8/334/s1, Table S1: Information of oil samples.
