# Supplementary material for: Phytosterol Contents of Edible Oils and Their Contributions to Estimated Phytosterol Intake in the Chinese Diet
Source: Foods. 2019 Aug 9;8(8):334. doi: 10.3390/foods8080334 (PMC6723959; doi:10.3390/foods8080334)
Supplement: Supplementary file 1 [file foods-08-00334-s001.pdf]

Phytosterol Contents of Edible Oils and Their Contribution to Estimated Phytosterols Intake in the Chinese Diet

Liangxiao Zhang*

[1] Oil Crops Research Institute, Chinese Academy of Agricultural Sciences, Wuhan 430062, China
[2] Key Laboratory of Biology and Genetic Improvement of Oil Crops, Ministry of Agriculture and Rural Affairs, Wuhan 430062, China
[3] Laboratory of Quality and Safety Risk Assessment for Oilseed Products (Wuhan), Ministry of Agriculture and Rural Affairs, Wuhan 430062, China
[4] Key Laboratory of Detection for Mycotoxins, Ministry of Agriculture and Rural Affairs, Wuhan 430062, China
[5] Quality Inspection and Test Center for Oilseed Products, Ministry of Agriculture and Rural Affairs, Wuhan 430062, China
***** Correspondence: Corresponding authors: Dr. Liangxiao Zhang at Oil Crops Research Institute, Chinese Academy of Agricultural Sciences, Wuhan 430062, China Tel.: +86 27 86812943; Fax: +86 27 86812862 E-mail addresses: liangxiao_zhang@hotmail.com (L. Zhang)

Table S1. Information of oil samples.

Oil sample		Brand	Description	Producing area
Peanut oil	1	Red Dragonfly	Pressed peanut oil	Chongqing
	2	Luhua	Peanut oil	Yantai City, Shandong Province
	3	Hope Full	First grade pressed peanut oil	Langfang City, Hebei Province
	4	Leyouyou	First grade pressed peanut oil	Dongying City, Shandong Province
	5	Chubao	Peanut oil	Guangzhou City, Guangdong Province
	6	Daomai	Pure peanut oil	Shenzhen City, Guangdong Province
	7	Laoxiang	Mild pressed peanut oil	Shenzhen City, Guangdong Province
	8	Fine Life	First grade peanut oil	Linyi City, Shandong Province
	9	Arawana Grandmother Township	Mild pressed peanut oil	Shanghai
	10	Runji	First grade peanut oil	Shanghai
Soybean oil	1	Red Dragonfly	First grade soybean oil	Chongqing
	2	Yingma	Soybean oil	Zhongshan City, Guangdong Province
	3	Lotus	Soybean oil	Guangzhou City, Guangdong Province
	4	Arawana	Soybean oil	Shanghai
	5	Arawana	Soybean oil	Shanghai
	6	Jinye	Soybean oil	Zhenjiang City, Jiangsu Province
	7	Fulinmen	Soybean oil	Tianjin
	8	Hope Full	First grade refined soybean oil	Langfang City, Hebei Province
	9	Zhongan	Cooked soybean oil	Heilongjiang Province
	10	Lotus	Soybean oil	Guangzhou City, Guangdong Province
	11	Gold Ingots	Soybean oil	Guangzhou City, Guangdong Province

	12	AKA	First grade soybean oil	Nanjing City, Jiangsu Province
	13	Linlong	Soybean oil	Wuhan City, Hubei Province
	14	Zhongchang	Soybean oil	Wuhan City, Hubei Province
Rapeseed oil	1	Xiancan	Pure yellow rapeseed oil with special flavor	Chengdu City, Sichuan Province
	2	Xiancan	First grade pressed rapeseed oil	Chengdu City, Sichuan Province
	3	Huixiangsui	Non-transgenic rapeseed oil	Chengdu City, Sichuan Province
	4	Fulinmen	Rapeseed oil	Suzhou City, Jiangsu Province
	5	Chulaixiang	Rapeseed oil	Hangzhou City, Zhejiang Province
	6	Ruyi	Rapeseed oil	Huzhou City, Zhejiang Province
	7	Evergrand Khingan	Green rapeseed oil	Hohhot, Inner Mongolia Autonomous Region
	8	Guchuan	Rapeseed oil	Beijing
	9	Panzhongcan	Pure rapeseed oil	Linxiang City, Hunan Province
	10	Knife	Rapeseed oil with low erucic acid	Shenzhen City, Guangdong Province
	11	Longquanshan	Pure pressed rapeseed oil	Deyang City, Sichuan Province
	12	Laoxiang	Mild pressed rapeseed oil	Shenzhen City, Guangdong Province
	13	Evergrand Khingan	Green rapeseed oil	Cuihua City, Heilongjiang Province
	14	Great Value	Rapeseed oil	Zunyi City, Guizhou Province
Sesame oil	1	Yusheng	Sesame oil	Fuling District, Chongqing
	2	Lovely Day	Sesame oil	Tianjin
	3	Xiang Wang	Pure sesame oil	Chengdu City, Sichuan Province
	4	Santian	Sesame oil	Minhang Area, Shanghai
	5	Yangshi	Sesame oil	Shaoxing City, Zhejiang Province
	6	Totole	Sesame oil	Zhumadian City, Hena Province
	7	Arawana	Sesame oil	Wuhan City, Hubei Province
	8	Lvbao	Sesame oil	Beijing

	9	Ruifu	Sesame oil	Weifang City, Shandong Province
	10	Haitian	Pure sesame oil	Foshan City, Guangdong Province
	11	Longquanshan	Pure sesame oil	Mianyang City, Sichuan Province
	12	Yanzhuang	Pure sesame oil	Hefei City, Anhui Province
	13	Lee Kum Kee	Sesame oil	Jiangmen City, Guangdong Province
	14	Great Value	Pure sesame oil	Shanghai
	15	Blessing Mill	Black sesame oil	Wuhan City, Hubei Province
Olive oil	1	Ybarra	Extra virgin olive oil	Spain
	2	Bertolli	Extra virgin olive oil	Italy
	3	Betis	Extra virgin olive oil	Spain
	4	Bargallo	Extra virgin olive oil	Spain
	5	Olivoila	First cold pressed olive oil	the Mediterranean
	6	Kniser Lena Mest	Olive blend oil	Nanchang City, Jiangxi Province
	7	Agric	Extra virgin olive oil	Greece
	8	Evergrand Khingan	Extra virgin olive oil	Spain
	9	Dintel	Extra virgin olive oil	Spain
	10	Rhino	Extra virgin olive oil	Spain
	11	Mueloliva	Extra virgin olive oil	Spain
	12	Tree Life	Extra virgin oilve oil	Australia
	13	Great Value	100% extra virgin oilve oil	Spain
	14	Olitalia	Extra virgin olive oil	Italy
Camellia oil	1	Sunplan	Camellia oil	Ningde City, Fujian Province
	2	Sunplan	Camellia oil for the women who are in confinement	Ningde City, Fujian Province
	3	jinjian	Original ecology clod-pressed camellia oil	Liuyang City, Hunan Province
	4	Jinshibenxiang	Camellia oil	Yichun City, Jiangxi Province

	5	Oil-tea camellia oil	Camellia oil	Shanghai
	6	Qiandaoyuan	Camellia oil	Hangzhou City, Zhejiang Province
	7	Arawana	Camellia oil	Shanghai
	8	Jinshibenxiang	Camellia oil	Yichun City, Jiangxi Province
	9	First Kitvhen	Camellia oil	Shangrao City, Jiangxi Province
	10	Qiyunshan	Camellia oil	Qiyunshan, Jiangxi Province
	11	Runxin	Organic camellia oil	Yichun City, Jiangxi Province
Sunflower oil	1	Fine Life	First grade sunflower oil	Suzhou City, Jiangsu Province
	2	Pietro Coricell	Sunflower oil	Italy
	3	Richevo	sunflower seed oil	Spain
	4	Meilin	Sunflower oil	Handan City, Hebei Province
	5	Westerner	First grade pressed sunflower oil	Lanzhou City, Gansu Province
	6	Luhua	Squeezing sunflower seed oil	Hohhot, Inner Mongolia Autonomous Region
	7	Runji	First grade pressed sunflower oil	Shanghai
	8	Fulinmen	First grade pressed sunflower oil	Bengbu City, Anhui Province
	9	Abrilsol	Refined sunflower oil	Spain
	10	Yuchushanpin	sunflower seed oil	Hohhot, Inner Mongolia Autonomous Region
	11	Dhenp	Refined sunflower oil	Ukraine
	12	Longevity Flower	Fragrant sunflower seed oil	Binzhou City, Shandong Province
	13	Great Value	Sunflower seed oil	Nantong City, Jiangsu Province
	14	Evergrand Khingan	Sunflower seed oil	Nantong City, Jiangsu Province
	15	Mighty	Sunflower seed oil	Hohhot, Inner Mongolia Autonomous Region
Corn oil	1	Wesson	Corn oil with cholesterol free	America
	2	Zhongchang	Corn oil rich in tocopherol	Wuhan City, Hubei Province
	3	Arowana	Corn oil rich in sterols	Wuhan City, Hubei Province

	4	Runji	Pressed corn oil	Shanghai
	5	Fulinmen	Pressed corn oil rich in sterols	Huanggang City, Hubei Province
	6	Xiwang	Physical pressed corn oil	Zouping City, Shandong Province
	7	Arowana	Pressed corn oil with 8000 ppm sterols	Wuhan City, Hubei Province
	8	Fulinmen	Pressed corn oil rich in sterols	Zhangjiagang City, Jiangsu Province
	9	Carepal	King corn oil	Siping City, Jilin Province
	10	Guchuan	First grade pressed corn oil	Beijing
	11	Wannianxing	Pressed corn oil	Heilongjiang Province
	12	Luhua	Squeezing corn oil	Chongqing
	13	Fufeng	First grade pressed corn oil	Linyi City, Shandong Province
	14	Knife	Corn oil	Shenzhen City, Guangdong Province
	15	Zhongchang	Corn oil	Hubei Province
Rice oil	1	Costadoro	Rice bran oil	Italy
	2	Olitalia	Rice bran oil	Italy
	3	Jinwang	Rice bran oil	Laohekou City, Hubei Province
	4	Arowana	Rice bran oil with 3000 ppm oryzanol	Shenzhen City, Guangdong Province
	5	Costadoro	Rice bran oil with gamma oryzanol	Italy
	6	Longevity Flower	Rice embryo oil	Binzhou City, Shandong Province
	7	Jinwang	Preseed rice bran oil	Laohekou City, Hubei Province
Flaxseed oil	1	Aidu	Flaxseed oil	Zhangjiakou City, Hebei Province
	2	Dinghe	Flaxseed oil rich in essential nutrients	Inner Mongolia Autonomous Region
	3	Chengrun	flaxseed oil	Ningxia
	4	Benchi	Cold-pressed dewaxed flaxseed oil	Inner Mongolia Autonomous Region
	5	Kunhua	100% pure flaxseed oil	Anyang City, Henan Province
	6	Grandma Kitchen	Flaxseed oil	Inner Mongolia Autonomous Region

	7	Mengguxiang	Cold-pressed dewaxed flaxseed oil	Inner Mongolia Autonomous Region
	8	Xinqidian	Flaxseed oil	Zhangjiakou City, Hebei Province
	9	Yipinhong	First grade pressed flaxseed oil	Jinchang City, Gansu Province
	10	Yuchushanpin	flaxseed oil	Inner Mongolia Autonomous Region
Walnut oil	1	Nouriz	Physical pressed walnut oil	France
	2	Kangbangmeiwei	Wild walnut oil	Yunnan
	3	La Tourangelle	Roasted walnut oil	California
Peony seed oil	1	Ruipu	Peony seed oil	Tongling City, Anhui Province
	2	Ren Tang	Peony seed oil	Tongling City, Anhui Province
	3	Nanyuanguose	Extraction of valuable peony seed oil	Changzhou City, Jiangsu Province
Grapeseed oil	1	Sosafty	First grade grapeseed oil	Gaomi City, Shandong Province
	2	Jingsenlikang	100% Pure grapeseed oil	Anyang City, Henan Province
	3	La masfia	Grapeseed oil	Spain
